# Holographic Sensor Based on Bayfol HX200 Commercial Photopolymer for Ethanol and Acetic Acid Detection

**DOI:** 10.3390/s23218776

**Published:** 2023-10-27

**Authors:** Ioana-Adriana Potărniche, Julia Marín-Sáez, M. Victoria Collados, Jesús Atencia

**Affiliations:** 1 Basis of Electronics Department, Faculty of Electronics, Telecommunication and Information Technology, University of Cluj-Napoca, 400114 Cluj-Napoca, Romania; 2 Applied Physics Department, Escuela Politécnica Superior, University of Zaragoza, Crta. de Cuarte s/n, 22071 Huesca, Spain; 3 Applied Physics Department, Engineering Research Institute of Aragon (I3A), Faculty of Science, University of Zaragoza, Pedro Cerbuna 12, 50009 Zaragoza, Spain; vcollado@unizar.es

**Keywords:** holographic sensors, acetic acid sensor, ethanol sensor, photopolymer, reflection holograms

## Abstract

This paper presents a holographic sensor based on reflection holograms recorded in the commercial photopolymer Bayfol^®^ HX 200. The recording geometry and index modulation of the hologram were optimised to improve accuracy for this specific application. The sensor was subjected to tests using various analytes, and it exhibited sensitivity to acetic acid and ethanol. The measurements revealed a correlation between the concentration of the analyte in contact with the sensor’s surface and the resulting wavelength shift of the diffracted light. The minimum detectable concentrations were determined to be above 0.09 mol/dm^3^ for acetic acid and 5% (*v*/*v*) for ethanol. Notably, the sensors demonstrated a rapid response time. Given that ethanol serves as a base for alcoholic beverages, and acetic acid is commonly found in commercial vinegar, these sensors hold promise for applications in food quality control.

## 1. Introduction

Holography is an optical technique that entails recording an interference pattern created by the superposition of two coherent electromagnetic waves on a light-sensitive material [1]. This interference generates an intensity pattern that induces alterations in either the material’s refractive index or thickness (referred to as phase holograms) or in its absorption properties (known as absorption holograms).

Holographic gratings are formed by the interference of two plane waves. When these waves reach the photosensitive material from opposite sides, a reflection hologram is generated, and the recorded fringes are nearly parallel to the material’s surface. Conversely, if the waves are incident on the same side of the material, a transmission hologram is created, with fringes oriented near-perpendicular to the surface. Reflection gratings exhibit higher chromatic selectivity and can diffract narrower spectrums than transmission gratings [2], which makes them well-suited for sensing applications that require a narrow spectral width.

Furthermore, if the intensity pattern induces changes in the material’s refractive index or thickness, the diffraction efficiency can reach 100%. The diffraction efficiency and the wavelength of the diffracted spectrum depend on the angle of illumination (angular selectivity), as well as the overall thickness of the holographic material and the recording geometry.

Holographic sensors are devices that undergo changes in their optical properties in response to the presence of an analyte or external stimuli [3,4]. Variations in refractive index or grating spacing can lead to alterations in diffracted intensity or wavelength shifts. These sensors offer several advantages over other optical sensors, making them well-suited for a wide range of applications.

One key advantage is their lightweight nature, and they do not require additional power supplies, making them ideal for use as disposable sensors [5,6]. Moreover, they eliminate the need for specialised personnel or complex equipment for data acquisition and interpretation. Smartphone applications can automate the process of data acquisition, interpretation, and simplified result delivery [7].

Holographic sensors find widespread use in various fields, including environmental monitoring [8,9], medical diagnosis [5,10,11,12], and bacterial analysis [6,13]. To cater to the specific demands of these applications, the photosensitive material employed in these sensors is often a self-processing material that can be customised accordingly.

Naydenova et al. [8] developed a self-processing photopolymer composed of a blend of two monomers, a polymeric binder, a photosensitizer, and a co-initiator. The deposited layer thickness of this sensor ranged from 30 to 180 μm. This particular sensor was designed for measuring humidity and temperature.

Oliveira et al. [5] presented a Ugi-holographic sensor specifically developed for the detection of cocaine. This sensor incorporated a Ugi ligand immobilised on a hydrogel layer, serving as the receptor. The sensitivity of the sensor was assessed using xenobiotic drugs, and the findings indicated its ability to detect concentrations of cocaine exceeding 36 mM.

Bhatta et al. [6] introduced four self-prepared holographic sensors, each designed for different applications. Among them is a methacrylic acid-based photopolymer employed for pH sensing, a sensor utilizing 2-(bis-carboxymethyl-amino)-6-(2-metyl-acryloylamino)-hexanoic acid for metal ion detection, and two other sensors based on casein and starch. The use of self-processed materials for bacterial spore detection offers several advantages, such as immunity to particle impurities and the capability to detect viable spores, making it a preferred method over alternative techniques.

Liu et al. [14,15] utilised a polymer hydrogel as the recording material for holographic sensors capable of detecting the pH of a solution within a specific range (from 4 to 8).

Yu et al. [10] presented a holographic sensor modified with beta-cyclodextrin (β-CD) for detecting ibuprofen in urine and plasma, serving clinical and forensic applications.

Davies et al. [11] achieved glucose detection using hydrogel-based, reusable holographic sensors.

The aforementioned papers employed reflection holograms, where a change in the angle of illumination resulted in a different colour displayed to the user, along with a variation in efficiency. This could potentially lead to errors in analyte detection, as the effect may resemble the sensor’s response to the presence of the analyte. However, none of the cited works analysed or optimised the recording geometry to account for this effect.

Furthermore, the previously mentioned papers used laboratory-made polymer-recording materials that required chemical fabrication, which can be cumbersome and dependent on batch processing. Adopting a commercial recording material could simplify the preparation of recording samples. However, commercial materials have limited options regarding characteristics such as thickness and spectral sensitivity range. Therefore, prior investigation is necessary to obtain the desired reconstruction behaviour.

This paper introduces a holographic sensor based on phase reflection holograms recorded in the commercial Bayfol HX200 photopolymer [16]. The optimal geometry for recording and reconstructing the reflection hologram is discussed to enhance detection accuracy. The detection capability has been examined for various analytes, with a focus on ethanol and acetic acid, both of which are relevant in food quality control applications. Furthermore, there are no other photopolymer based holographic sensors reported in the literature for the detection of these analytes.

## 2. Theoretical Model for Reflection Holograms

**Figure 1:**
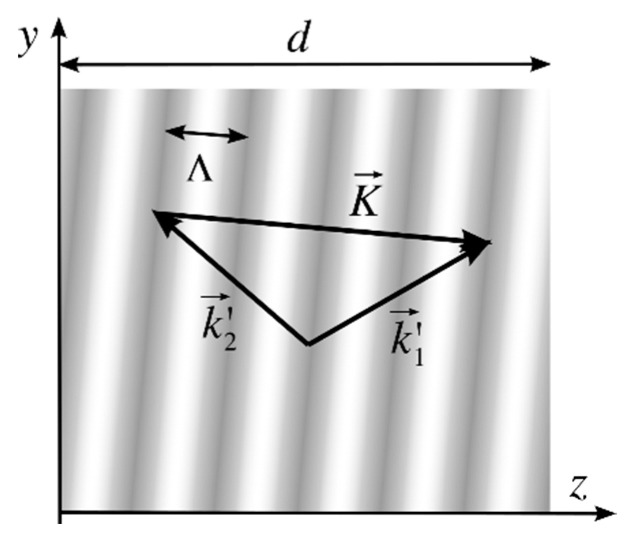
Volume reflection grating with recording wavevectors $\vec{k_{1}^{\prime }},\;\vec{k_{2}^{\prime }}$ into the medium and grating vector $\vec{K}$.

Volume holograms are theoretically described using Kogenik’s Coupled-Wave Theory [2]. For a volume reflection hologram two orders of diffraction are present: the transmitted wave that corresponds to the 0 order and the diffracted wave that corresponds to order +1. The refractive index profile recorded in a volume phase hologram can be expressed as

(1) $$n\left(\vec{r}\right)=n_{a}+∆n\cdot sin\left(\vec{K}\left(\vec{r}\right)\cdot \vec{r}\right),$$

where *n_a_* is the average refractive index, Δ*n* is the amplitude of the index modulation, and $\vec{K}$ is the grating vector that is perpendicular to the planes of constant refractive index (marked with grey levels in Figure 1). The module of the grating vector depends on the spatial period of the grating, Λ,

(2) $$K=\frac{2\pi }{\Lambda },$$

The grating vector can be calculated as the difference between the two construction wavevectors $k_{1}^{\prime }$ and $k_{1}^{\prime }$ inside the recording material, as shown in Figure 1.

(3) $$\vec{K}=\vec{k_{1}^{\prime }}-\vec{k_{2}^{\prime }}.$$

The construction wavevectors lie onto the y-z plane, so they can be expressed as

(4) $$\vec{k_{1}^{\prime }}=\frac{2\pi }{\lambda_{R}}n_{a\;}\left(m_{1}^{\prime }\vec{u_{y}}+n_{1}^{\prime }\vec{u_{z}}\right),$$

(5) $$\vec{k_{2}^{\prime }}=\frac{2\pi }{\lambda_{R}}n_{a\;}\left(m_{2}^{\prime }\vec{u_{y}}+n_{2}^{\prime }\vec{u_{z}}\right),$$

where *λ_R_* is the recording wavelength, and $m_{1}^{\prime }$, $n_{1}^{\prime }$, $m_{2}^{\prime }$, and $n_{2}^{\prime }$ are the direction cosines of the waves inside the medium. Replacing Equations (4) and (5) in (3) the grating vector can be calculated as

(6) $$\vec{K}=\frac{2\pi }{\lambda_{R}}n_{a\;}\left[\left(m_{1}^{\prime }-m_{2}^{\prime }\right)\vec{u_{y}}+\left(n_{1}^{\prime }-n_{2}^{\prime }\right)\vec{u_{z}}\right].$$

In the reconstruction stage of the hologram, a monochromatic wave with a wavelength *λ* that can be defined by the following wavevector is considered.

(7) $$\vec{k_{0}^{\prime }}=\frac{2\pi }{\lambda }n_{a\;}\left(m_{0}^{\prime }\vec{u_{y}}+n_{0}^{\prime }\vec{u_{z}}\right).$$

The diffracted wave will have a wavevector, $\vec{k_{+1}^{\prime }}$,

(8) $$\vec{k_{+1}^{\prime }}=\frac{2\pi }{\lambda }n_{a\;}\left(m_{+1}^{\prime }\vec{u_{y}}+n_{+1}^{\prime }\vec{u_{z}}\right).$$

**Figure 2:**
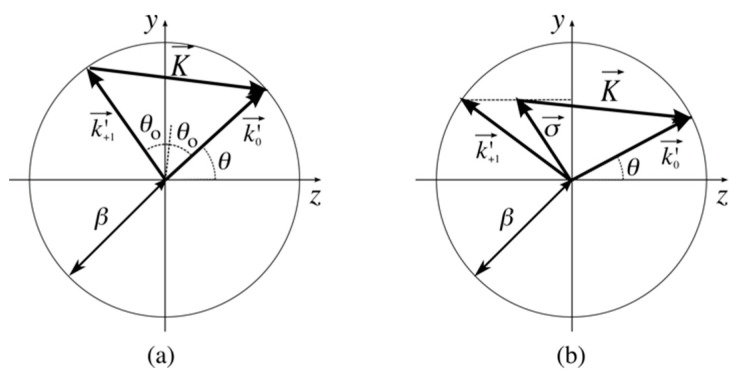
Wave vectors $\vec{k_{0}^{\prime }},\;\vec{k_{+1}^{\prime }}$ and grating vector $\vec{K}$ when (**a**) the Bragg condition is fulfilled and (**b**) when it is not fulfilled.

The maximum energy transferred to the diffracted wave $\vec{k_{+1}^{\prime }}$ happens when

(9) $$\vec{k_{+1}^{\prime }}=\vec{k_{0}^{\prime }}-\vec{K},$$

which corresponds to the so-called Bragg condition that can be obtained geometrically from Figure 2a,

(10) $$\lambda =2\Lambda n_{a}sin\theta_{0},$$

where $\theta_{0}$ is the semi-angle depicted in that figure.

When Bragg condition is not fulfilled (Figure 2b), the diffracted wave is given by the projection of $\vec{k_{+1}^{\prime }}$, $\vec{k_{0}^{\prime }}$, and $\vec{K}$ on the hologram plane, *z* = 0 [1],

(11) $$\vec{k_{+1}^{\prime }}\cdot \vec{u_{y}}=(\vec{k_{0}^{\prime }}-\vec{K})\cdot \vec{u_{y}}.$$

From Equation (11), $m_{+1}^{\prime }$ and $n_{+1}^{\prime }$ can be calculated as

(12) $$m_{+1}^{\prime }=m_{0}^{\prime }-\mu \left(m_{1}^{\prime }-m_{2}^{\prime }\right),$$

(13) $$n_{+1}^{\prime }=\sqrt{1-{m_{+1}^{\prime }}^{2}},$$

where *μ* is defined as

(14) $$\mu =\frac{\lambda }{\lambda_{R}}.$$

The diffraction efficiency can be calculated with the following equations:

(15) $$\eta ={\left[1+\frac{1-\frac{\xi^{2}}{\nu^{2}}}{{\left[sinh\;\left(\nu^{2}-\xi^{2}\right)\right]}^{2}}\right]}^{-1},$$

(16) $$\xi =\frac{\vartheta d}{2n_{+1}^{\prime }},$$

(17) $$\vartheta =\frac{\beta^{2}-{\left|\vec{\sigma }\right|}^{2}}{2\beta },$$

(18) $$\nu =\frac{\pi d\Delta n}{\lambda \sqrt{n_{0}^{\prime }\;n_{+1}^{\prime }}},$$

where $\beta =\frac{2\pi }{\lambda }n_{a}$, $\vec{\sigma }=\vec{k_{0}^{\prime }}-\vec{K}$ (Figure 2b), and parameter *ϑ* evaluates the deviation from the Bragg condition. It can be rewritten as a function of the direction cosines of the recording and illumination waves,

(19) $$\vartheta =\frac{1}{2}\beta \left\{1-{\left[m_{0}^{\prime }-\mu \left(m_{1}^{\prime }-m_{2}^{\prime }\right)\right]}^{2}-{\left[n_{0}^{\prime }-\mu \left(n_{1}^{\prime }-n_{2}^{\prime }\right)\right]}^{2}\right\}.$$

The value of *ν* is determined by the index modulation Δ*n* obtained in the recording step.

With expressions (15) to (19), it is possible to calculate efficiency for a reconstruction wave with any wavelength or direction, which allows the simulation of the performance of any recording geometry.

## 3. Holographic Sensor Design

**Figure 3:**
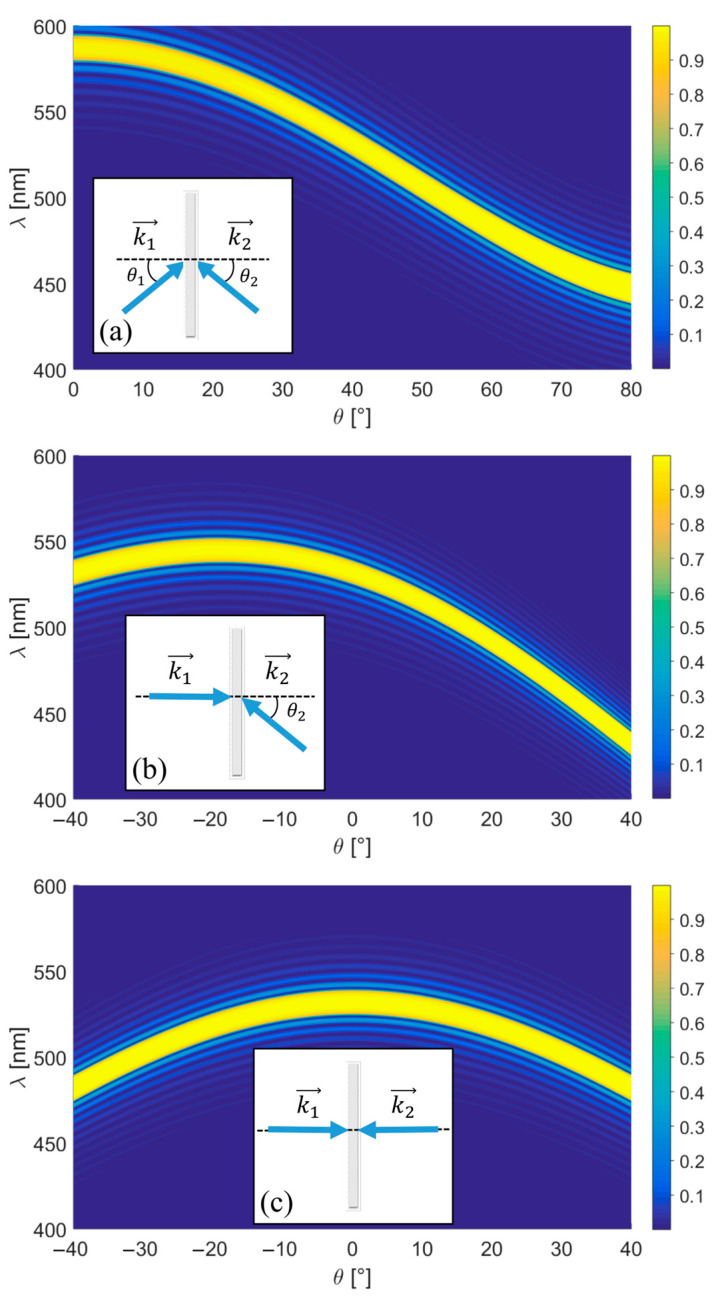
Recording geometry (reference and object wave angles in air: (**a**) *θ*_1_ > 0° and *θ*_2_ < 0°; (**b**) *θ*_1_ = 0° and *θ*_2_ < 0°; (**c**) *θ*_1_ = *θ*_2_ = 0°) and efficiency depending on the reconstruction wavelength *λ* and replay incidence angle. Blue arrows represent the wavevectors.

As described in Section 2, the recording of a reflection hologram can be performed using different directions of the recording waves, each of which determines angular and chromatic selectivity. In order to determine the optimal geometry for recording a reflection hologram for use in a sensing application, three representative recording geometries (Figure 3a–c) are analysed, with the corresponding recording angle values presented in Table 1. It should be noted that other values derived from these three geometries yielded similar results without affecting the subsequent reasoning. The simulations have been carried out using MATLAB software (version R2021a, The Mathworks, Inc., Natick, MA, USA). 

Figure 3 shows the simulation results of each geometry when the hologram is reconstructed at different angles of incidence *θ* (Figure 2) and wavelengths. The simulation is performed using the following parameters: recording wavelength *λ_R_* = 532 nm, photopolymer thickness *d* = 16 μm (corresponding to Bayfol HX 200 photopolymer used in the experimental section), and *ν* = π for 532 nm (determined by Δ*n* as shown in Eqaution (18)). The maximum efficiency (indicated in yellow) is achieved when the Bragg condition is satisfied for a specific wavelength at each angle of incidence. For 532 nm, this occurs when reconstructing with either of the recording angles.

Holographic sensors work by absorbing an analyte solution, resulting in swelling and an increase in the grating period. Consequently, this leads to a shift of the diffracted wavelength towards longer wavelengths, as illustrated in Equation (10). To ensure a broader range of the visible spectrum for detecting this shift, it is desirable to achieve maximum efficiency at a shorter wavelength. This condition is satisfied for normal incidence with geometry (c). The measurement range could be expanded by recording holograms using a laser with a shorter wavelength. However, as will be demonstrated in Section 4.2, recording at 532 nm provides a suitable measurement range for this study.

In general, small changes in the angle of incidence result in changes in the wavelength that satisfies the Bragg condition. This effect is minimised when the angle of incidence meets the Bragg condition for the longest possible wavelength. As illustrated in Figure 3, this corresponds to 0° for geometries (a) and (c) and −20° for geometry (b). In these cases, minor reconstruction angle changes cause negligible shifts in the wavelength that fulfils the Bragg condition, making the sensor less sensitive to small misalignments.

Furthermore, illuminating the hologram perpendicularly is the most practical approach for easy alignment, particularly in sensing applications where analyte detection is not performed in a lab setting.

Therefore, considering all these factors, the optimal recording geometry for reflection holographic sensors is (c) of Figure 3. All further analysis in this paper is carried out with this configuration.

**Figure 4:**
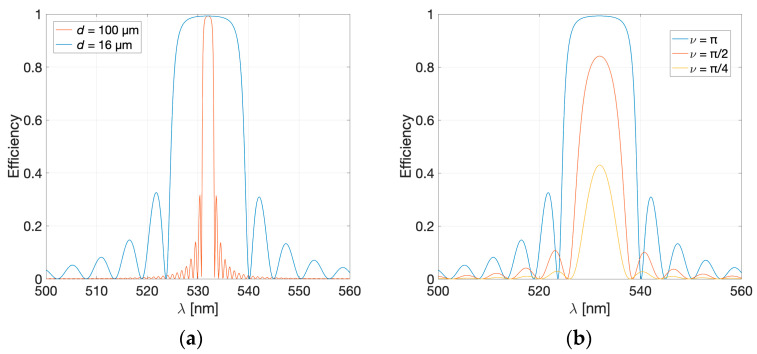
Efficiency of a reflection hologram (recording geometry of Figure 3c) reconstructed at perpendicular incidence, (**a**) with different photopolymer thicknesses (16 μm with blue and 100 μm with orange), and *ν* = π; and (**b**) with 16 μm photopolymer thickness for *ν* = π (blue), *ν* = π/2 (orange) and *ν* = π/4 (yellow).

Figure 4a (Blue line) shows the calculated efficiency vs. reconstruction wavelength of the chosen hologram (Figure 3c) when illuminated with perpendicular incidence only. As the peak is rather broad, a small shift would not be noticed. Thus, a narrower peak is desirable. There are three parameters that can be varied to obtain narrower peaks with the chosen geometry: thickness of the photopolymer, index modulation (*ν* parameter) and recording wavelength.

Figure 4a (orange line) shows the calculated efficiency that would be obtain for a thickness of 100 μm, as opposed to the 16 μm used so far. The simulations were performed using *ν* = π for both photopolymers. The 16 μm photopolymer has a peak width of approximately 15 μm, whereas the width is five times smaller for the 100 μm photopolymer.

Another approach to achieve narrower peaks is by varying the value of the ν parameter in Equation (18). Figure 4b illustrates the simulated efficiency of a reflection hologram recorded on a 16 μm-thick photopolymer for three different values of the ν parameter: π, π/2, and π/4. The results demonstrate that the width of the diffracted peaks is dependent on the value of ν. Higher values of ν (such as ν = π for 532 nm) correspond to broader peaks with maximum efficiency. Conversely, as the values of ν decrease, the peaks become narrower, but the efficiency also decreases. Reduction in the ν parameter leads to a significant decrease in efficiency (achieving efficiencies of 14% for ν = π/8 and 3.8% for ν = π/16, respectively), making it challenging to identify the peak. Through simulations, we have determined that a ν parameter of π/4 provides a favourable balance between efficiency and peak width, as depicted in Figure 4b. If the efficiency of the hologram is not critical for the intended application, as is the case in this study, thin photopolymers such as Bayfol HX200 are suitable for recording the holographic sensor.

The diffraction peaks can be narrower if the recording wavelength is lower than 532 nm. Bayfol HX200 is sensitive to a range of wavelengths between 420 nm and 680 nm, so this would be possible, although more powerful lasers or longer exposure times would be necessary as the photopolymer sensitivity decreases towards 420 nm.

**Figure 5:**
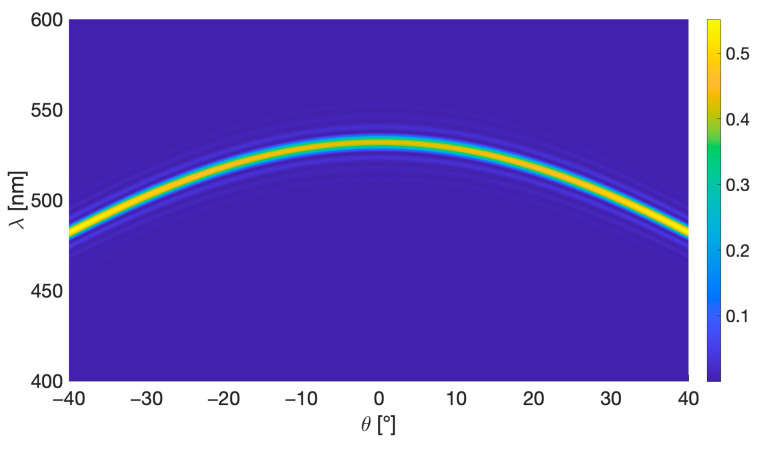
Efficiency of a reflection hologram (geometry of Figure 3c: *θ*_1_ = *θ*_2_ = 0°, *ν* = π/4, 16 μm photopolymer thickness) as a function of both the reconstruction wavelength (λ) and the replay incidence angle.

Figure 5 shows the simulation results obtained for a photopolymer thickness of 16 μm, values of *ν* = π/4, recording angles of *θ*_1_ = *θ*_2_ = 0°, and a recording wavelength of 532 nm. This combination has been selected for the sensors in this study.

## 4. Experimental Results

The selected material for recording holographic sensors in this study is the commercial photopolymer Bayfol HX200 [17], as mentioned earlier. Photopolymers are materials composed of monomers that undergo a series of chemical changes when exposed to light. These monomers combine and form polymers, resulting in areas with different concentrations of molecules in the material structure when exposed to light. As a consequence of these concentration differences, a modulation of the refractive index occurs.

### 4.1. Calibration of Bayfol HX200

Holograms can be recorded in Bayfol HX200 using light with a wavelength between 440 nm to 680 nm. The technical datasheet [18] specifies that no wet or thermal post-recording procedures are required. The photopolymer thickness is 16 μm ± 2 μm.

**Figure 6:**
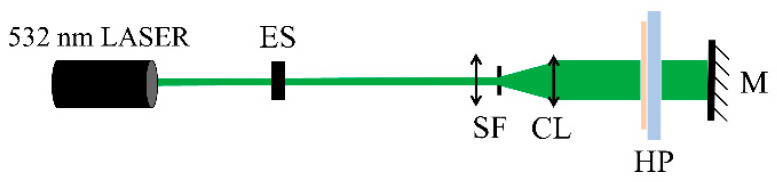
Recording setup: ES—electronic shutter; SF—spatial filter; CL—collimating lens; HP—holographic plate; and M—mirror.

To ensure mechanical stability during recording, samples of Bayfol HX200 (23 × 70 mm) were laminated onto microscope slides before being exposed to laser radiation. The slides were previously cleaned with distilled water to eliminate any dust or other particles. The recording setup, shown in Figure 6, used a 532 nm CW laser (DPSS Oxxius LMX, Lannion, France). One of the two recording beams was directed onto the holographic plate and passed through it, whereas the other beam was reflected off a mirror and directed onto the opposite side of the plate to constitute the second recording beam. The diameter of the exposed zone is around 2 cm.

After the recording of the holographic gratings, the photopolymer is let to rest for five minutes in a black box. Subsequently, the sample is photocured using a white LED lamp for 25 min [13].

**Figure 7:**
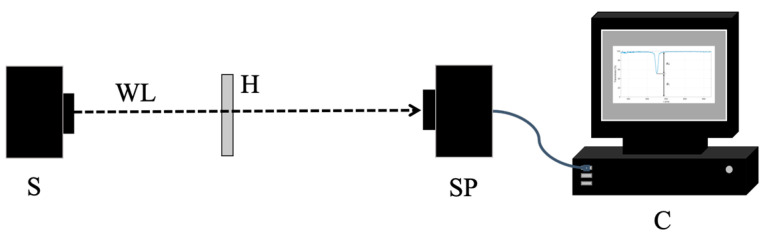
Setup for transmission spectrum measurement: S—Tungsten Halogen Lamp; WL—collimated white light; H—reflection hologram; SP—spectrophotometer; and C—computer.

**Figure 8:**
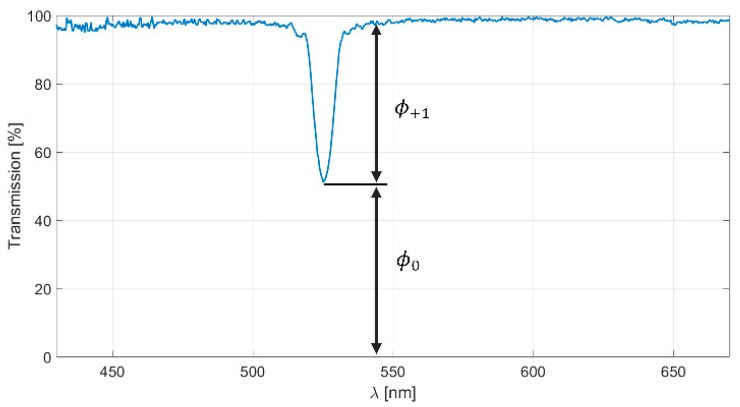
Transmission spectrum (blue line) for a reflection hologram.

The holograms were illuminated at a reconstruction angle of 0° using an LS-1 Tungsten Halogen Lamp (360 nm–2500 nm) from Ocean Optics (Duiven, The Netherlands), as shown in Figure 7. The diameter of the illumination beam at the hologram plane is around 5 mm. The transmission spectra of the holograms were acquired using the USB2000 VIS-NIR-ES spectrophotometer (Ocean Optics, Duiven, The Netherlands), which covers the wavelength range from 350 to 1000 nm. Figure 8 shows a typical transmission spectrum obtained for a reflection hologram.

The relative efficiency of the hologram depends on the flux of the transmitted wave $\left(\phi_{0}\right)$ for the order 0 and the flux of the diffracted wave $\left(\phi_{+1}\right)$ and can be calculated using

(20) $$\eta =\frac{\phi_{+1}}{\left(\phi_{+1}+\phi_{0}\right)}.$$

**Figure 9:**
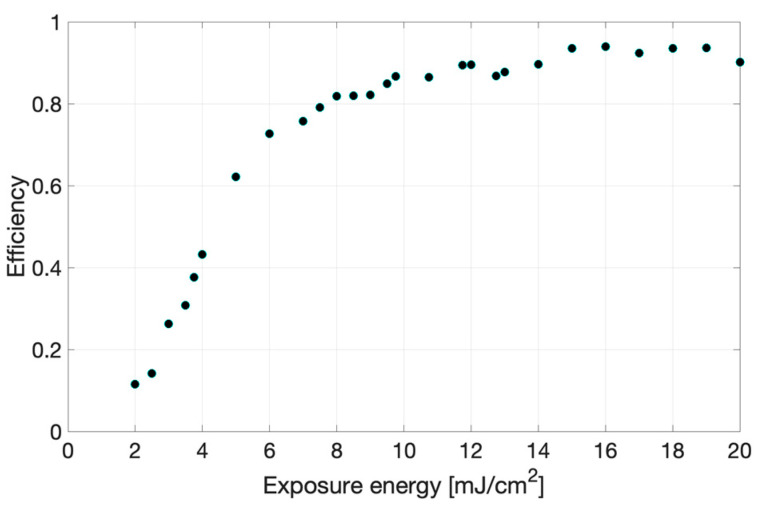
Efficiency of reflection gratings recorded in Bayfol HX200 versus exposure energy.

The relative efficiency values obtained for the reflection holograms recorded on the Bayfol^®^ HX200 are presented in Figure 9 as a function of exposure energy. The recording time varies from 1.19 s to 11.97 s (for laser intensities around 1 mW/cm^2^). The efficiency increases with the exposure energy between 0–10 mJ/cm^2^, and, afterwards, its value remains around 0.9 (higher efficiency values are not obtained due to Δ*n* saturation effects [19]). To obtain results shown in Figure 5, corresponding to *ν* = π/4, the exposure energy must be around 4 mJ/cm^2^.

### 4.2. Holographic Sensor Results

**Figure 10:**
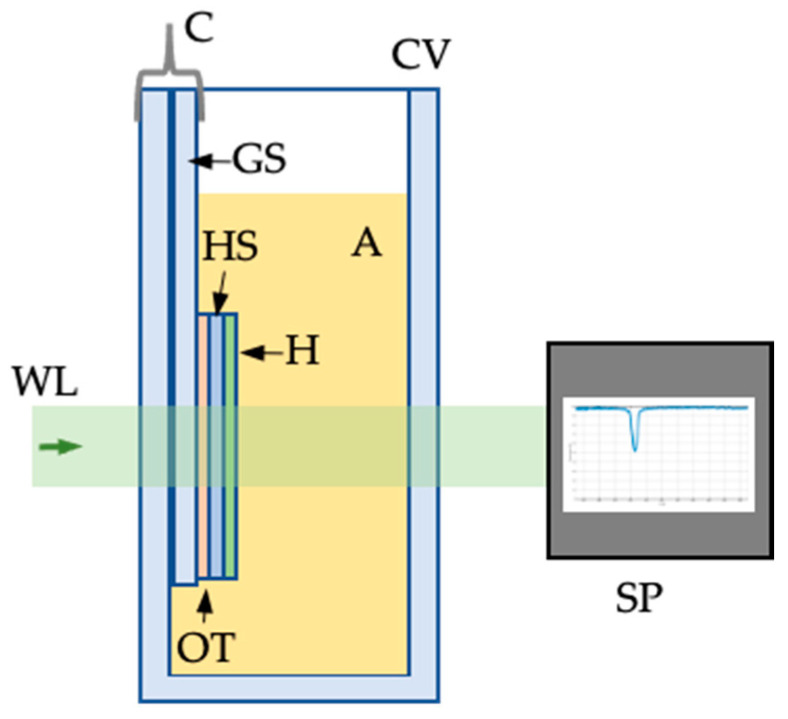
Setup for sensing: WL—collimated white light; CV—cuvette for analyte; GS—glass support; OT—optical tape; HS—hologram substrate; H—reflection hologram (photopolymer); A—analyte solution; C—clamp; SP—spectrophotometer.

Several holograms were recorded with an exposure energy of 4 mJ/cm^2^, as explained in Section 4.1. After recording, each hologram was detached from the glass support, flipped, and the substrate was glued with optical tape (Thorlabs optically clear double-sided adhesive tape) on the glass support. The glass support was fixed into an optical glass cuvette (15 × 30 × 50 mm) with a clamp, as shown in Figure 10, so that the hologram was illuminated perpendicularly with white light, and the photopolymer is directly exposed to the analyte solution with enough rigidity to measure the transmittance with the spectrophotometer.

The transmitted beam reached the spectrophotometer (which had a precision of 0.3 nm) and was monitored with a computer. An analyte solution was poured in the cuvette, and the transmission spectrum was stored every minute up until five minutes.

The transmitted beam had a minimum that corresponds to a maximum in the diffracted beam. A wavelength shift (Δ*λ*) was observed due to the presence of the analyte and was calculated as the difference between the wavelength of the minimum transmission peak obtained for the hologram without the analyte and that of the hologram in contact with the analyte.

Various analytes (ethanol; sulfuric, acetic, ascorbic, and citric acid; and sodium hydroxide) were used to test the sensibility of the holographic sensor. All solutions were prepared using distillate water, for which the holographic sensor showed no response.

First of all, ethanol was tested. Twenty-two samples with ethanol concentrations ranging from 5% (*v*/*v*) up to 100% (*v*/*v*) were prepared. The uncertainty in concentration was lower than 0.5%.

**Figure 11:**
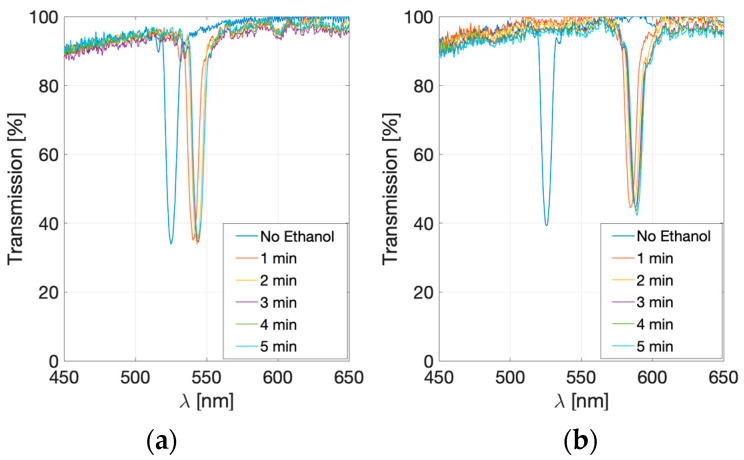
Transmission spectra of hologram when it is in contact with 20% (**a**) and 50% (*v*/*v*) (**b**) ethanol solutions from 0 to 5 min.

Figure 11 represents the transmission spectra when the analyte is a 20% (*v*/*v*) ethanol solution (left) or a 50% one (right). These spectra reveal wavelength shifts of the holographic peaks according to the quantity of ethanol that comes in contact with the surface of the sensor. The 20% (*v*/*v*) solution has a wavelength shift of 15.5 nm after five minutes of analyte exposure, whereas, for the 50% (*v*/*v*) solution, the wavelength shift increases up to 58.7 nm. As the shift is similar between 1 and 5 min, the value corresponding to 1 min will be taken as the measurement value.

**Figure 12:**
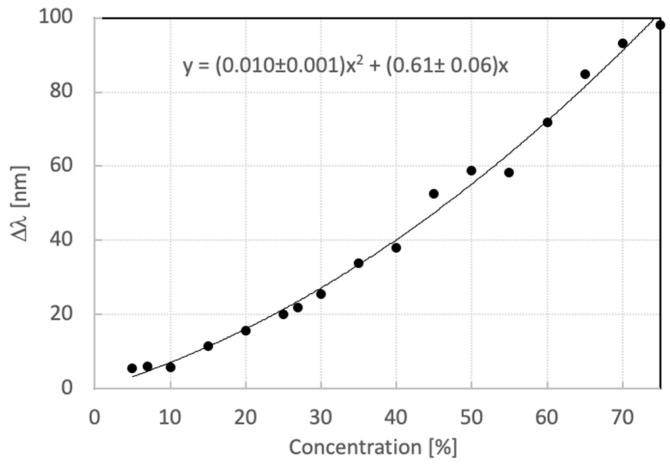
Fitting curve for ethanol concentration detection.

The fitting curve that would define the relation between the ethanol concentration and the wavelength shift for concentrations from 0 to 75% is described by a quadratic equation y = Ax^2^ + Bx, with A = 0.010 ± 0.001 nm and B = 0.61 ± 0.06 nm. The R^2^ coefficient calculated is 0.9929. The quadratic fit is represented in Figure 12. This fitting could also be appropriate for concentrations higher than 75% and shorter analyte exposure times (Appendix A). Nevertheless, higher concentrations may not be relevant for food quality control applications. Concentrations lower than 5% do not trigger a significant response of the sensor.

**Figure 13:**
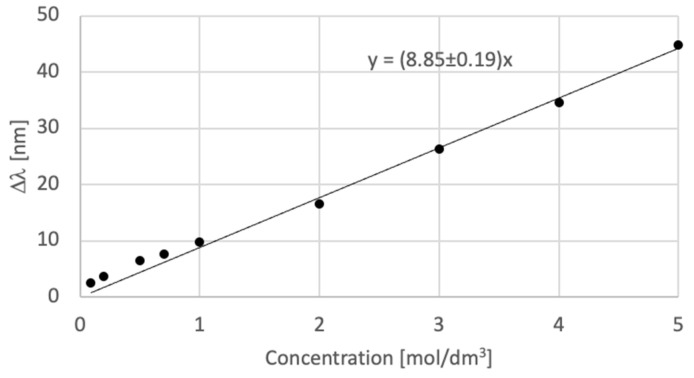
Fitting curve for acetic acid.

The measurements of the holographic sensor response for acetic acid concentrations show that as the molarity increases the wavelength shift increases as well. Nine solutions with molarities from 0.09 ± 1 × 10^−4^ mol/dm^3^ to 5 ± 3 × 10^−3^ mol/dm^3^ were used for the measurements. The wavelength shifts obtained after 5 min of exposure to acid (which provides maximum response) are shown in Figure 13. The results during the five-minute analysis of the holograms are included in Appendix A. For molarities lower than 0.09 mol/dm^3^, the holographic sensor based on Bayfol HX200 is not sensitive to acetic acid.

The fitting curve that would define the relation between the molarity of the acetic acid and the wavelength shift is described by a linear equation y = Ax, with A = 8.85 ± 0.19 nm·dm^3^/mol. The R^2^ coefficient calculated is 0.9964.

## 5. Discussion

Reflection holograms in Bayfol HX200 are found to be able to detect ethanol and acetic acid, but not other analytes (Table 2). Further tests were carried out with different organic acids (ascorbic and citric acid) and an inorganic acid and a base (sulfuric acid and sodium hydroxide), but no significant wavelength shift was obtained. Therefore, Bayfol HX200 holographic sensors are not able to detect the pH of a solution. Table 2 shows a summary of the results obtained.

The formulation and specific composition of the Bayfol HX200 material, once it is polymerized, have not been published, which makes it challenging to provide a detailed explanation of its possible interactions with each analyte. However, considering that Bayfol HX200 is an acrylate-based photopolymer, we hypothesise that the shift of the efficiency peak is primarily attributed to the diffusion mechanism of the analyte, rather than a chemical reaction or potential chemical bonding with the analytes. When holograms are only exposed to distilled water, no changes are observed. However, we propose that when the hologram is immersed in a solution containing an analyte, there is a concentration gradient that allows the analyte to diffuse into the polymeric grating, causing it to swell and consequently shift the diffraction peak. The efficiency of this diffusion process is influenced by the molecular size of the analyte. This phenomenon should explain why the holograms are sensitive to ethanol and acetic acid, which have a similar structure, consisting of two carbon atoms, whereas other analytes with similar chemical characteristics, such as ascorbic acid or citric acid, which have much larger molecules, cannot diffuse through the spaces within the polymer lattice that forms the hologram.

Furthermore, we can conclude that the diffusion process occurs uniformly throughout the thickness of the hologram, ensuring that the grating period remains consistent across the material thickness. If the analyte molecules were to spread unevenly, the grating period would vary, resulting in an increased width of the diffraction peak after shift. However, this effect was not observed in the experimental measurements.

The reason why they are not sensitive to sodium hydroxide or sulfuric acid may be because they are inorganic compounds that dissociate into ions that either do not diffuse into the polymer or do not cause polymer swelling.

When the analyte is removed, the sensor does not regain its initial thickness, an effect that is especially noticeable for high concentrations. This is because a permanent deformation of the polymer matrix remains. Therefore, these sensors cannot be considered reversible and, as a result, are not reusable.

Future work could include new sensing experiments with different analytes, in particular other alcohols such as methanol or isopropanol, to better study the working mechanism of the holographic sensor. Additionally, the recording of reflection holograms with diffusing objects [19], which would allow a visual detection of the wavelength shift (namely, with a colour change in the diffracted light), could be considered. That way, no optical equipment (spectrophotometer) would be necessary for the analyte detection stage.

## 6. Conclusions

A holographic sensor was developed for the detection of two different analytes. The sensor consists of a reflection hologram recorded in the commercially available acrylate-based photopolymer Bayfol HX200, which has a thickness of 16 μm. One of the significant advantages of using a commercial photopolymer in holographic sensors is the simplified pre-recording process, eliminating the need for chemical knowledge or additional chemical products to prepare the photopolymer.

Simulations based on Kogelnik’s Coupled Wave Theory were conducted, revealing that for sensors based on reflection holograms, the optimal configuration is achieved by recording the holograms with beams perpendicular to the plane of the hologram. This configuration allows for a broad range of detection wavelengths and minimises potential alignment errors. It has been demonstrated that the 16 μm commercial photopolymer can be effectively used for the intended application when holograms are recorded with an efficiency of around 40%, resulting in sufficiently narrow chromatic selectivity peaks.

Tests were conducted on the holographic sensor to evaluate its ability to detect analytes. It exhibited accurate detection capabilities for solutions of acetic acid and ethanol. For acetic acid, concentrations up to 5 mol/dm^3^ and a contact time of 5 min resulted in a wavelength shift Δ*λ* of about 45 nm. A linear dependence of Δ*λ* with molarity was observed. Regarding ethanol, it was observed that concentrations up to 75% and a contact time of 1 min induced a wavelength shift Δ*λ* of approximately 100 nm. The relationship between concentration and shift followed a quadratic dependence.

Furthermore, holographic sensors based on Bayfol HX200 were also tested with solutions of distilled water and different acids (sulfuric, ascorbic, and citric acid) and a base (sodium hydroxide) to establish their sensitivity regarding pH. The results show that there is no significant wavelength shift for solutions with pH ranging from 1.3 to 12, as shown in Table 2. Therefore, these holographic sensors are not able to detect the pH of a solution.

These holographic sensors have potential applications in food quality control, as ethanol is present in alcoholic beverages, and acetic acid is a component of commercial vinegar. Their rapid response time and ease of data interpretation make them suitable for use by non-experts in the fields of holography or sensor technology. Further investigations are required to understand how the system would behave in more complex sample matrices compared to simple aqueous solutions containing only a single analyte.

## Figures and Tables

**Table 1:** Numerical values of the recording angles in air for the three geometries in Figure 3.

	(a)	(b)	(c)
*θ*_1_ (°)	40	0	0
*θ*_2_ (°)	−40	−40	0

**Table 2:** Detection range for studied analytes.

Analyte	Detection Time	Detection Range
Ethanol	1 min	5% to 75%
Acetic acid	5 min	0.09 to 5 mol/dm^3^
Citric acid	5 min	Unresponsive up to 2.5 mol/dm^3^ (pH = 1.3)
Ascorbic acid	5 min	Unresponsive up to 2.8 mol/dm^3^ (pH = 1.4)
Sulfuric acid	5 min	Unresponsive up to 0.005 mol/dm^3^ (pH = 2)
Sodium hydroxide	5 min	Unresponsive up to 0.01 mol/dm^3^ (pH = 12)

**Figure A1:**
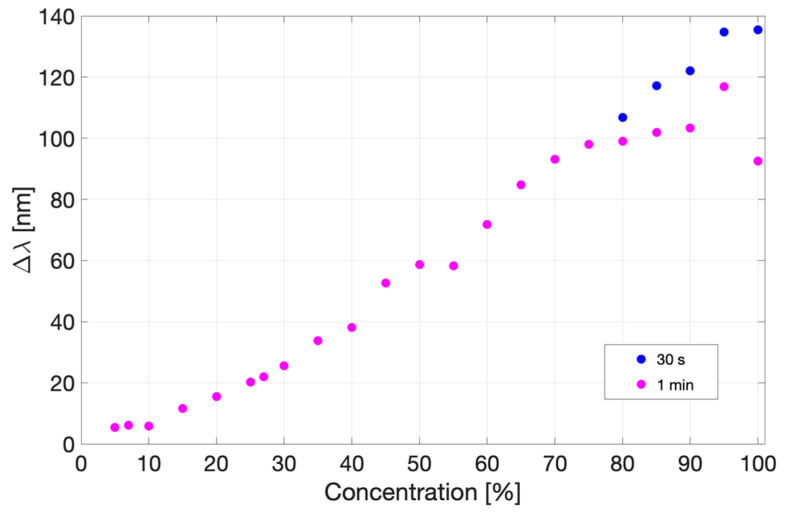
Wavelength shift for 5–100% (*v*/*v*) solutions of ethanol.

**Figure A2:**
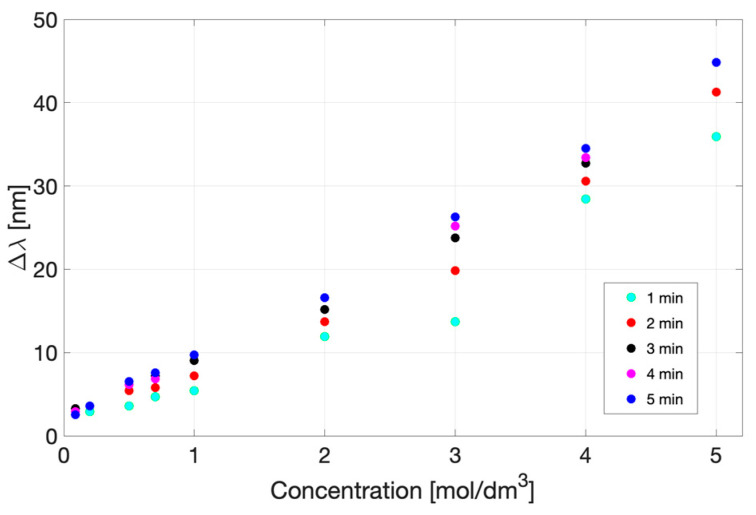
Wavelength shift vs. acetic acid concentration during the five-minute analysis.

## Data Availability

Data are contained within the article or Appendix A.

## Appendix A. Holographic Sensor Response to Additional Concentrations and Contact Times

For ethanol concentrations greater than 75% (*v*/*v*), the analyte is absorbed faster, and the maximum wavelength shift is obtained after 30 s of exposure to the alcohol. After that time, the wavelength shift is towards shorter wavelengths. Figure A1 shows the values obtained for all ethanol solutions (with purple dots the values obtained after one minute of measurement and with blue dots the measurement performed after 30 s for high concentrations only).

The wavelength shift of the diffracted spectrum of the holographic sensor when in contact with acetic acid after every minute during the analysis is shown in Figure A2. The holographic sensor response to this analyte is slower than to ethanol, and highest after five minutes.

## References

[1] Syms R. (1990). Practical Volume Holography.

[2] Kogelnik H. (1969). Coupled wave theory for thick hologram gratings. Bell Syst. Tech. J..

[3] Naydenova I. (2020). Holographic Sensors. Optical Holography. Materials, Theory and Applications.

[4] Davies S., Hu Y., Jiang N., Blyth J., Kaminska M., Liu Y., Yetisen A.K. (2021). Holographic Sensors in Biotechnology. Adv. Funct. Mater..

[5] Oliveira N.C.L., El Khoury G., Versnel J.M., Moghaddam G.K., Leite L.S., Lima-Filho J.L., Lowe C.R. (2018). A holographic sensor based on a biomimetic affinity ligand for the detection of cocaine. Sens. Actuators B Chem..

[6] Bhatta D., Christie G., Madrigal-González B., Blyth J., Lowe C.R. (2007). Holographic sensors for the detection of bacterial spores. Biosens. Bioelectron..

[7] Moghaddam G.K., Lowe C.R. (2017). Smartphone-based quantitative measurements on holographic sensors. PLoS ONE.

[8] Naydenova I., Jallapuram R., Toal V., Martin S. (2009). Characterisation of the humidity and temperature responses of a reflection hologram recorded in acrylamide-based photopolymer. Sens. Actuators B Chem..

[9] Mikulchyk T., Walshe J., Cody D., Martin S., Naydenova I. (2017). Humidity and temperature induced changes in the diffraction efficiency and the Bragg angle of slanted photopolymer-based holographic gratings. Sens. Actuators B Chem..

[10] Yu J., Gai Z., Cheng J., Tian F., Du K., Wei W., Li Y., Gao Q., Zou C., Qian R. (2023). Construction of beta-cyclodextrin modified holographic sensor for the determination of ibuprofen in plasma and urine. Sens. Actuators B Chem..

[11] Davies S., Hu Y., Blyth J., Jiang N., Yetisen A.K. (2023). Reusable Dual-Photopolymerized Holographic Glucose Sensors. Adv. Funct. Mater..

[12] Davies S., Hu Y., Jiang N., Montelongo Y., Richardson A., Blyth J., Yetisen A.K. (2022). Reversible photonic hydrogel sensors via holographic interference lithography. Biosens. Bioelectron..

[13] Bhatta D., Christie G., Blyth J., Lowe C.R. (2008). Development of a holographic sensor for the detection of calcium dipicolinate—A sensitive biomarker for bacterial spores. Sens. Actuators B Chem..

[14] Liu H., Yu D., Zhou K., Wang S., Luo S., Li L., Wang W., Song Q. (2018). Novel pH-sensitive photopolymer hydrogel and its holographic sensing response for solution characterization. Opt. Laser Technol..

[15] Liu H., Wang R., Yu D., Luo S., Li L., Wang W., Song Q. (2019). Direct light written holographic volume grating as a novel optical platform for sensing characterization of solution. Opt. Laser Technol..

[16] Bruder F.-K., Fäcke T., Rölle T. (2017). The Chemistry and Physics of Bayfol® HX Film Holographic Photopolymer. Polymers.

[17] Marín-Sáez J., Atencia J., Chemisana D., Collados M.-V. (2016). Characterization of volume holographic optical elements recorded in Bayfol HX photopolymer for solar photovoltaic applications. Opt. Express.

[18] Covestro Deutschland AG (2020). Bayfol HX200 Datasheet. https://solutions.covestro.com/-/media/covestro/solution-center/products/datasheets/imported/bayfol/bayfol-hx200_en_86194384-20033146-20033738.pdf.

[19] Vázquez-Martín I., Marín-Sáez J., Gómez-Climente M., Chemisana D., Collados M.-V., Atencia J. (2021). Full-color multiplexed reflection hologram of diffusing objects recorded by using simultaneous exposure with different times in photopolymer Bayfol® HX. Opt. Laser Technol..

